# Effect of Abnormal Savda Munziq, a Traditional Uighur Herbal Medicine, on Pulmonary Function and Aquaporins of COPD Rat Model with Abnormal Savda Syndrome

**DOI:** 10.1155/2017/7176263

**Published:** 2017-05-29

**Authors:** Gao Zhen, Halmurat Upur, Wang Jing, Jing Jing, Li Zheng, Xu Dan, Li Fengsen

**Affiliations:** National Clinical Research Base of Traditional Chinese Medicine, Traditional Chinese Medicine Hospital Affiliated to Xinjiang Medical University, Urumqi 830000, China

## Abstract

**Objective:**

To investigate the effect of abnormal savda munziq (ASM) on the pulmonary function and expression of lung-specific aquaporins in the rat model of chronic obstructive pulmonary disease with abnormal savda syndrome (ASSCOPD).

**Methods:**

Eighty male rats were randomized into ASSCOPD, COPD, and control groups. ASSCOPD was further categorized into ASM and non-ASM groups. COPD model was established by combining fumigation with airway instillation of elastase; ASSCOPD model was developed based on COPD by induction with dry cold diet, cold dry environment, and plantar electric stimulation. ASM was administered twice daily. The pulmonary function was evaluated based on respiration. The mRNA and protein levels of AQPs were estimated by real-time PCR and Western blot, respectively.

**Results:**

MV, TV, the mRNA level of AQP5, and the protein expression of AQP1, AQP4, and AQP5 were increased in ASMCOPD compared to ASSCOPD.

**Conclusion:**

The pulmonary function was impaired in ASSCOPD group; the expression of AQP1, AQP4, and AQP5 was decreased at protein and mRNA levels in ASSCOPD group. ASM can improve the pulmonary function in ASSCOPD for MV and TV. ASM could elevate the protein expression of AQP1, AQP4, and AQP5 and the mRNA level of AQP5 in lung tissue.

## 1. Introduction

Chronic obstructive pulmonary disease (COPD) is a preventable and treatable respiratory disease characterized by airflow limitation; however, the restricted airflow cannot be completely reversed, and it continuously progresses if not effectively controlled. COPD was suggested to be associated with the abnormal inflammatory response of lung tissues to the cigarette smoke and other harmful gasses or particles. The incidence of COPD was reported as 8.2% in the cohort of 40-year-old or more senior populations in China. The data were collected from seven cities and provinces [1]; the incidence of COPD in South Korea was 13.4% from a similar cohort [2]. COPD had been considered to be one of the leading causes of disability and deaths globally, bringing enormous economic and social burden [3]. Therefore, the effective prevention and treatment of COPD continue to remain a vital public health concern to be resolved urgently.

However, the etiology and pathogenesis mechanism of COPD are not completely elucidated, and the current medication cannot effectively alleviate the long-term deteriorating pulmonary function [4]. Thus, seeking an efficient therapeutic method from traditional Uighur medicine might be valuable. COPD was classified into four types according to the traditional Uighur medicine principle, including abnormal savda syndrome (ASS), abnormal blood, abnormal phlegmatic temperament, and abnormal choleric temperament. ASS is the most common type (40.64%) with a severe condition and unfavorable prognosis, being prone to deterioration [5].

The ASS COPD required ASS treatment based on the traditional Uighur medicine principle, and the representative prescription abnormal savda munziq (ASM) is a common compound prescription for complicated diseases [6]. ASM is a traditional Uighur herbal compound prepared in accordance with the traditional medicine principle, from Mizani Tibiye (law of medicine) compiled by an ancient expert of Uighur medicine, Mohammad Akbar Elzani. ASM is composed of ten different medicines including cordia fruit, tongue grass, maidenhair fern, dracocephalum, lavender, fennel, jujube, licorice, humifusa, and stab sugar [7].

Aquaporins (AQPs) are a family of small transmembrane proteins that aid in the accelerated movement of water, selectively and bidirectionally through lipid bilayers [8, 9]. In addition to contributing to the water permeability of the cell membrane, AQPs participate in nerve signal transduction, skin flexibility, fat metabolism, membrane permeability to gasses, and cell migration and proliferation [10]. AQPs are composed of several forms in the lung: AQP1 is found in the peribronchiolar, alveolar endothelium, and visceral pleura; AQP3 is in the trachea; AQP4 is in the airway epithelia and the trachea, and AQP5 is at the apical membrane of type I alveolar epithelial cells [11]. Differential expression of AQP1 and AQP5 is one of the critical parameters reflecting pulmonary injury and microcirculation alterations [12]; the expression of AQP5 could be mechanistically involved in COPD pathogenesis or expression inflammatory response [13]. Currently, there is a lack of studies on the correlation between AQP and ASS COPD defined by traditional Uighur medicine, though AQPs play critical roles in the pathogenesis of COPD. Therefore, in the present study, the effect of ASM on ASS COPD model was examined, and the regulation of the expression of AQPs was investigated, which might be one of the therapeutic mechanisms of ASM.

## 2. Material and Methods 

### 2.1. Animals

Eighty clean-grade male rats, 5 weeks old, with body weight of 150 ± 20 g were bought from the Experimental Animal Center of Xinjiang Medical University. The production license number was SCXK (Xin) 2003-0001. The rats were adaptively raised for 3 days at the center, with free access to water and forage. The study was approved by the Ethics Department of Experimental Animal Center, Xinjiang Medical University.

### 2.2. Medicine

ASM was originally derived from Mizani Tibiye (law of medicine) compiled by a famous ancient expert of Uighur medicine, Mohammad Akbar Elzani. It was composed of 10 herbs including lavender, fennel, cordia fruit, jujube, bugloss, dracocephalum, licorice, maidenhair fern, humifusa, and stab sugar. Free decoction particles were purchased from Jiang Yin Tianjiang Pharmaceutical Co., Ltd. (Jiangsu, China).

### 2.3. Instruments and Reagents

Artificial climate chamber (FLI-2000H) was obtained from EYELA (Japan). The respiratory function experimental platform (BUXCO MA1320) was procured from Buxco (USA). The electronic balance (BS-1105) was bought from Beijing Sartorius AG. The microscope (DM600B-1) was from Leica (Germany), and the biological transmission electron microscope (JEM-1230) was from Electronics Co. (Japan). The acrylic exposure box was assembled in the group (length, width, and height were 60, 70, and 100 cm, resp.) with a vent of 2.0 cm diameter at the top, and the smoke was evenly spread by an internally suspended electronic fan driven by CPU before air drying with 250 g silica gel desiccant. The cigarettes (Hardmen) were from Shandong Zhong Cigarette Co., Ltd. (China) with 11 mg tar, 0.8 mg nicotine, and 13 mg carbon monoxide. Elastase was obtained from Shanghai Aladdin biochemical Technologies Inc., 30 U/mg (Batch number: H1326040).

### 2.4. Grouping and Model Rat Preparation

The rats were classified into four groups by random number table method, including COPD with ASS group (ASSCOPD, *n* = 40), COPD group (*n* = 20), and control group (*n* = 20).

*COPD Group [14]*. The rats were treated with elastase at day 30 after the initiation of the experiment, at a dose of 20 U elastase (solubilized in 0.8 mL normal saline) per 100 g body weight through tracheal instillation. The rats were treated daily with cigarettes' smoke from days 1 to 29 and days 31 to 90, 2 cigarettes/day (20 rats were placed in one box treated with the combustion of 20 cigarettes, which lasted for 1 h every morning and evening). The rats were raised at room temperature controlled at 25 ± 3°C and relative humidity of approximately 60–80%.

*ASSCOPD Group*. The rats were further treated based on the COPD group with the following modeling factors [15]. The rats were raised in the artificial climate box for 10 h every day, with the systematic temperature fixed at 6 ± 2°C from days 1 to 97 every night and relative humidity at about 25–32.8%. The rats were fed with free access to home-made feeds composed of conventional feeds, barley, and coriander at a ratio of 7 : 1.5 : 1.5, respectively. Moreover, the chronic stress was induced by intermittent plantar electric shock for 20 min/day (output voltage was ranged between 20 and 30 V and altered irregularly with the interval 0.2–20 min). All the modeling factors were continued for 97 days. 

*Control Group*. The rats were raised at a regulated temperature of 25 ± 3°C and relative humidity of about 60–80%. The animals were treated by tracheal instillation using an equivalent volume of normal saline that was used for the COPD group since day 30.

All the rats were raised for 97 days, and the ASSCOPD group was further divided into ASMCOPD group (treated with gavage of ASM for 7 days) and ASSCOPD group (treated with gavage of equivalent volume of normal saline for 7 days). The rats from all the groups were examined by noninvasive pulmonary functionality assessment and subsequently sacrificed by blood drawing; the tissue samples were obtained and preserved for further examination. 

*Tracheal Instillation of Elastase*. Surgical instruments were sterilized before operation by autoclave (121°C, 120 kPa, and 30 min). The rats were weighed individually and intraperitoneally injected with 0.4% pentobarbital sodium at a dose of 50 mg/kg body weight. The success of anesthesia was defined as no response to claw tip clamping. The rats were fixed onto the plates, and the 16-gauge casing needle was slowly inserted into trachea along the tongue. The needle was withdrawn and connected with 1 mL syringe. The rats were placed in a dorsally elevated position at 45°; half a volume of the solution was injected slowly when the rats were tilted to the left and another half when tilted to the right. Then, 1 mL air was injected when the position of rats was restored in order to inject all the remaining solution into the lung. The rats were patted slightly at the back so that the elastase solution could be evenly distributed in the lung. The rats were moved back to the original cases when the consciousness was regained, and the body temperature was monitored continuously during the whole process.

### 2.5. Interference Methods

ASMCOPD group was treated with Uighur medicine through gavage. The dose was calculated according to the “equivalent dose table for human and animal translation by body surface area” [16]. The daily dosage of ASM for a human with a body weight of 70 kg was 30 g, and according to the table, the corresponding daily dose for a rat with a body weight of 200 g was 0.54 g. A 5 mL ASM solution was administrated through gavage twice a day, each morning and evening, and the rats from the ASSCOPD, COPD, and control groups were treated with an equivalent volume of normal saline for 7 days.

### 2.6. Assessment Parameters and Methods

The general phenotype observations were recorded including physical activities, appearance, hair, the oral and nasal secretions, and respiratory tract-specific symptoms such as cough and asthma.

The pulmonary functions were assessed using noninvasive pulmonary functionality test system (Buxco) to measure the respiratory frequency (*F*), minute ventilation (MV), peek of inspiratory flow (PIF), peak of expiratory flow (PEF), inspiratory time (*T*_i_), expiratory time (*T*_e_), tidal volume (TV), enhanced pause (Penh), pause (PAU), and 50% tidal volume expiratory flow (EF50). The assessment was carried out according to Edgar et al. [17]. 

*Rat Pulmonary Pathological Observation*. The rats were sacrificed by drawing blood from inferior vena cava under anesthesia with pentobarbital sodium on days 90 and 97, respectively. The lung tissues were removed immediately including the middle lobe of the right lung, part of the trachea, and bronchus, which were all fixed in 10% formalin. The tissue specimen slices for pathological examination were processed by routine dehydration, embedded in paraffin, and sliced at a thickness of 2 *μ*m. Three consecutive slides prepared from each tissue block were subjected to HE staining for observing the histological morphology under the microscope. 

*Real-Time qPCR*. The levels of AQP1 mRNA, AQP4 mRNA, and AQP5 mRNA in lung tissue were measured by real-time qPCR. 40 cycles of each reaction were carried out, and the Ct value of AQP1, AQP4, and AQP5 was standardized by that of *β*-actin. The expression level of AQP1 mRNA, AQP4 mRNA, and AQP5 mRNA was calculated by the 2^−ΔΔCt^ method.

**Table:** 

	Upstream primer	GACTACACTGGCTGTGGGATCAA	
AQP1			115 bp
	Downstream primer	CCAGGGCACTCCCAATGAA	
			
	Upstream primer	AGGCAATGTGTGCACTGCTCTA	
AQP4			120 bp
	Downstream primer	AAGGTGTCAACGTCACACAACAA	
			
	Upstream primer	CATGGTGGTGGAGTTAATCTTGA	
AQP5			161 bp
	Downstream primer	CATGGAACAGCCGGTGAAGTAG	
			
	Upstream primer	GGAGATTACTGCCCTGGCTCCTA	
*β*-Actin			150 bp
	Downstream primer	GACTCATCGTACTCCTGCTTGCTG	

*Western Blot Analysis*. The total proteins of rat lung tissues were extracted in the RIPA lysis buffer. 20 *μ*g proteins were resolved by 12% SDS-PAGE after determining the protein concentration by the BCA method. The proteins were transferred to a membrane at 100 V for 2 h following electrophoresis at 120 V for 1 h. The membrane was blocked with 5% skim milk in TBST for 1 h, followed by incubation with goat anti-rat monoclonal antibodies against AQP1, AQP4, and AQP5, respectively, at 4°C overnight (all 1 : 200), and detected by rabbit anti-rat IgG secondary antibody (1 : 1000) after incubation at room temperature for 1 h. The membranes were further treated with ECL developer and visualized as well as analyzed by Bio-Rad software for grey scale analysis. The relative expression level of AQP1, AQP4, and AQP5 was estimated by normalizing against that of *β*-actin as the internal control.

### 2.7. Statistical Analysis

SPSS 11.5 software was utilized in the current study for statistical analysis. The results were presented as mean ± SD. The data fulfilled the normal distribution, and the homogeneity of variance was analyzed by ANOVA, and two-two comparison among multiple groups was processed by the LSD test. The two-two comparison was processed by Tamhane method for the data with the heterogeneity of variance. *P* < 0.05 was considered statistically significant.

## 3. Results

### 3.1. Pulmonary Functionality Comparison among Groups (Tables 1–4)

Compared to the control group, the value of *F*, MV, PIF, PEF, TV, and EF50 was decreased (*P* < 0.01), and the value of *T*_i_, *T*_e_, Penh, and PAU was increased (*P* < 0.01) in the COPD group. The value of *F*, MV, PIF, PEF, TV, and EF50 was decreased (*P* < 0.01), and the value of *T*_i_, *T*_e_, Penh, and PAU was increased (*P* < 0.01) in the ASSCOPD group. The value of *F*, MV, PIF, PEF, TV, and EF50 was decreased (*P* < 0.01), and the value of *T*_i_, *T*_e_, Penh, and PAU was increased (*P* < 0.01) in the ASMCOPD group.

Compared to the COPD group, the value of *F*, MV, and EF50 was decreased (*P* < 0.01, *P* < 0.05, and *P* < 0.01), and the value of *T*_i_, *T*_e_, TV, and Penh was increased (*P* < 0.01) in the ASSCOPD group; the value of F, EF50 was decreased (*p* < 0.01), and that of *T*_i_, *T*_e_, TV, and Penh was increased in the ASMCOPD.

Compared to the ASSCOPD group, the value of MV and TV was increased (*P* < 0.01) in the ASMCOPD group.

### 3.2. Pulmonary Pathological Examination Comparison among Groups (Figure 1)

The size of the alveolar wall was similar throughout the control group; the thickness of the alveolar wall was in the normal range without inflammatory cell infiltration or a small number of inflammatory cells scattered around (a). A large amount of inflammatory cell infiltration was observed in the COPD group (b). A large amount of inflammatory cell infiltration was observed in the ASSCOPD group (c). The inflammatory cell infiltration could be observed in ASMCOPD but was greatly improved in comparison with the COPD and ASSCOPD group (d).

### 3.3. mRNA Expression of AQP1, AQP4, and AQP5 in Lung Tissues of Rat Models (Table 5)

Compared to the control group, the mRNA level of AQP1 and AQP5 was decreased in the COPD group (*P* < 0.01) and the mRNA level of AQP1, AQP4, and AQP5 was decreased in the ASSCOPD group (*P* < 0.01), whereas the mRNA level of AQP1 was decreased in the ASMCOPD group (*P* < 0.01).

Compared to the COPD group, the mRNA level of AQP5 was increased in the ASMCOPD group (*P* < 0.05).

Compared to the ASSCOPD group, the mRNA level of AQP5 was increased in the ASMCOPD group (*P* < 0.05).

### 3.4. Protein Expression of AQP1, AQP4, and AQP5 in Lung Tissues of Rat Models (Figure 2, Table 6)

Compared to the control group, the protein expression of AQP1, AQP4, and AQP5 was decreased in the COPD group (*P* < 0.01) and the ASSCOPD group (*P* < 0.01), whereas the protein expression of AQP1 and AQP5 was decreased in the ASMCOPD group (*P* < 0.01).

Compared to the COPD group, the protein expression of AQP1, AQP4, and AQP5 was increased in the ASMCOPD group (*P* < 0.01).

Compared to the ASSCOPD group, the protein expression of AQP1, AQP4, and AQP5 was increased in the ASMCOPD group (*P* < 0.01).

## 4. Discussion 

Airway mucus hypersecretion led to the increased airway mucus viscosity and increased airway resistance, which adversely affected the clearance of mucus resulting in the residence and proliferation of pathogenic bacteria in the respiratory airway, aggravating the infection. This was a common characteristic of multiple chronic inflammatory airway diseases that was closely correlated with chronic bronchitis, asthma, bronchial dilation, and COPD [18]. The airway mucus hypersecretion was a significant clinical, pathological feature of COPD and considered as an independent risk factor for mortality and disease progression of COPD [19]. The increase in viscosity and acidic enhancement of mucus caused by airway hypersecretion altered the mucin structure which presented as significantly high glycosylation and sulfation on mucin. Moreover, irrespective of the onset [20] or stable period of COPD [21], the COPD patients with impaired pulmonary function had the goblet cell hyperplasia and increased expression of MUC5AC [22]. The mucus retention at the acute and late phase of COPD was correlated not only with the absolute increased amount of mucin but also with the unbalance between mucin and water or salt. The critical proteins that participated in the lung water transport process were AQPs. Both AQP1 and AQP5 played a crucial role in the water transport process driven by osmotic pressure in the lung tissue [23]. Additionally, AQP5 might be involved in the abnormal process of water transport [24], and decreased expression of AQP4 could reduce the water reabsorption capability of lung tissue [25]. Moreover, AQPs were also demonstrated to be involved in several pathophysiological processes, and AQP5 gene knockout could attenuate the bacteria clearance capability of the lung tissue [26]. In addition, AQP5 was one of the targeted genes leading to the airway hyperresponsiveness [27]. The expression of AQP4 was negatively correlated with the severity of inflammation of bronchial mucosa: the less AQP4 expressed, the more severity of airway inflammation [28]. The decreased expression of AQP5 was correlated with the increased expression of mucin in the COPD airway [29]. A similar negative correlation between AQP5 and MUC5AC was also found in the lung tissue of the asthma model of mouse [30]; the MUC5AC expression was significantly increased when AQP5 expression was downregulated by RNA interference [31]. Therefore, the differential expression of AQP1 and AQP5 was one of the critical parameters indicating the pulmonary microenvironment alteration by lung injury [32].

The main components of ASM include cordia fruit, tongue grass, and maidenhair fern. The cordia fruit could moisturize the lung and throat, relieve a cough, and reduce sputum. Therefore, it was used to treat dry and itchy throat, productive cough, thirst, aphonia, dysuria, and interference of defecation [33]. The tongue grass moisturized the tissues and was anti-inflammatory, relieving cough and asthma [34]. The maidenhair fern possessed the functions of anti-inflammation and detoxification, relieving cough and eliminating phlegm, mitigating asthma and pain. Therefore, it was commonly used for asthma, chest and lung viscosity disease, pneumonia, headache, chest pain, shortness of breath, asthma, long-term cough, sputum, and fever [35]. The extracts from maidenhair fern have been demonstrated to play a role in anti-inflammation, relieving cough and sputum, and mitigating asthma [36]. The terms of traditional medicine such as “moisturizing dryness syndrome” and “eliminating phlegm” were closely associated with the regulated distribution of “body fluids,” and the functions of “body fluids” were manifested as similar functions of mucin in airway [37]. Therefore, the effects and mechanisms of ASM on COPD model were investigated in the current study.

In the present study, the COPD model with ASS was successfully established. It was found that the pulmonary functions of COPD group and ASSCOPD group were similar regarding the tested parameters and developmental tendency; both groups manifested decreased respiratory volumes (MV and TV) and flows (PIF, PEF, and EF50) and increased *T*_i_, *T*_e_, Penh, and PAU. In addition, it was shown that the decreased *F*, MV, and EF50 significantly increased *T*_i_, *T*_e_, TV, and Penh in the ASSCOPD group. The treatment with ASM demonstrated an improved respiratory volume (MV and TV), though no significant effect was seen on the other parameters. Coupled with the AQPs' detection in the lung tissues, it was found that ASSCOPD had an even lower pulmonary function; the protein expression of AQPs (AQP1, AQP4, and AQP5) was lower than the other groups, and the mRNA level was also lower than the control group. Conversely, COPD presented decreased protein level of AQPs, whereas only the mRNA levels of AQP1 and AQP5 were reduced. Therefore, ASM treatment mainly increased the protein expression level of AQP1, AQP4, and AQP5 and only elevated the mRNA level of AQP5.

## 5. Conclusion

The lower pulmonary function was observed in the ASSCOPD model; AQP1, AQP4, and AQP5 had a decreased expression at both protein and mRNA levels. Uighur traditional medicine, ASM, could elevate the protein expression level of AQP1, AQP4, and AQP5 and only elevated the mRNA level of AQP5. Moreover, the MV and TV of ASSCOPD were improved, which might be one of the potential mechanisms of ASM for the treatment of COPD with ASS.

## Figures and Tables

**Figure 1:**
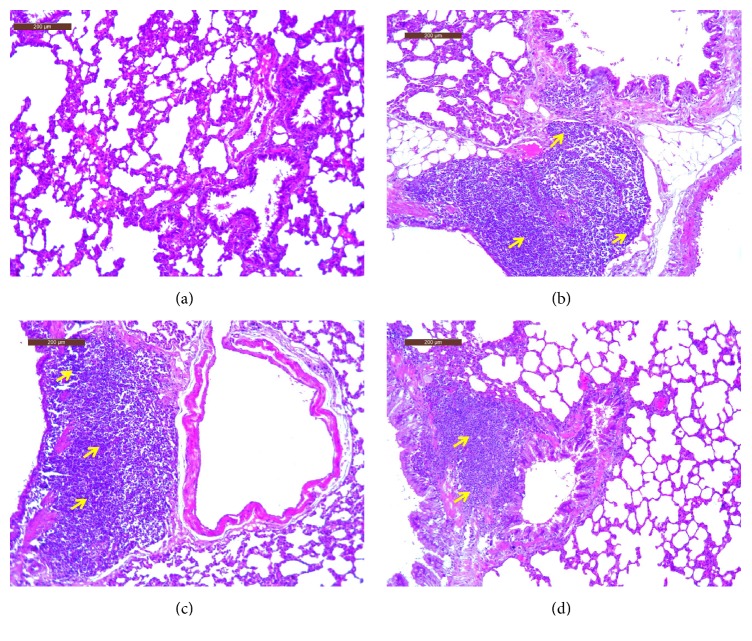
Pathological sections of lung tissue from rats of groups (HE staining, 200x). Note: inflammatory cell infiltration (yellow arrow).

**Figure 2:**
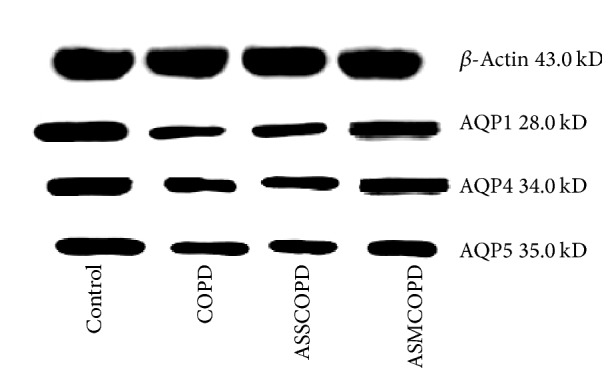
Comparison of AQP1, AQP4, and AQP5 protein expression in lung tissue among groups. Note: COPD: chronic obstructive pulmonary disease, ASSCOPD: COPD with abnormal savda syndrome, and ASMCOPD: ASSCOPD treated by abnormal savda munziq.

**Table 1:** Pulmonary functions *F* and MVB comparison among groups.

Group	*N*	*F* (bpm)	MV (mL)
COPD	10	145.42 ± 85.09^*※*^	168.95 ± 111.52^*※*^
ASSCOPD	10	132.12 ± 80.51^*※*▴▴^	159.59 ± 100.51^*※*▴*◆*^
ASMCOPD	9	127.82 ± 73.09^*※*▴▴^	167.75 ± 109.73^*※*^
Control	6	183.85 ± 106.58	320.59 ± 300.75
*F*		107.33	276.40

*Note*. ^*※*^Compared to the control group *P* < 0.01; ^▴^compared to the COPD group *P* < 0.05, ^▴▴^*P* < 0.01; compared to the ASMCOPD group ^*◆*^*P* < 0.01. *Note*. COPD: chronic obstructive pulmonary disease, ASSCOPD: COPD with abnormal savda syndrome, and ASMCOPD: ASSCOPD treated by abnormal savda munziq.

**Table 2:** Pulmonary functions PIFb and PEFb comparison among groups.

Group	*N*	PIF (mL/s)	PEF (mL/s)
COPD	10	11.32 ± 5.22^*※*^	8.53 ± 7.51^*※*^
ASSCOPD	10	10.95 ± 5.98^*※*^	8.58 ± 6.36^*※*^
ASMCOPD	9	11.43 ± 6.10^*※*^	8.95 ± 6.65^*※*^
Control	6	20.91 ± 17.22	15.23 ± 15.91
*F*		340.94	150.41

*Note*. ^*※*^Compared to the control group *P* < 0.01. *Note*. COPD: chronic obstructive pulmonary disease, ASSCOPD: COPD with abnormal savda syndrome, and ASMCOPD: ASSCOPD treated by abnormal savda munziq.

**Table 3:** Pulmonary functions *T*_i_ and *T*_e_ comparison among groups.

Group	*N*	*T*_i_ (s)	*T*_e_ (s)
COPD	10	0.19 ± 0.06^*※*^	0.35 ± 0.18^*※*^
ASSCOPD	10	0.21 ± 0.07^*※*▲^	0.38 ± 0.17^*※*▲^
ASMCOPD	9	0.21 ± 0.07^*※*▲^	0.38 ± 0.18^*※*▲^
Control	6	0.16 ± 0.07	0.30 ± 0.16
*F*		159.88	56.63

*Note*. ^*※*^Compared to the control group *P* < 0.01; ^▲^compared to the COPD group *P* < 0.01. *Note*. COPD: chronic obstructive pulmonary disease, ASSCOPD: COPD with abnormal savda syndrome, ASMCOPD: ASSCOPD treated by abnormal savda munziq.

**Table 4:** Pulmonary functions TV, Penh, PAU, and EF50 comparison among groups.

Group	*N*	TV (mL)	Penh	PAU	EF50 (mL/s)
COPD	10	1.25 ± 0.49^*※*^	0.70 ± 0.69^*※*^	0.88 ± 0.68^*※*^	0.58 ± 0.65^*※*^
ASSCOPD	10	1.33 ± 0.61^*※*▲*◆*^	0.82 ± 0.91^*※*▲^	0.94 ± 0.95^*※*^	0.51 ± 0.56^*※*▲^
ASMCOPD	9	1.40 ± 0.59^*※*▲^	0.84 ± 0.92^*※*▲^	0.93 ± 0.83^*※*^	0.51 ± 0.59^*※*▲^
Control	6	1.76 ± 1.23	0.55 ± 0.45	0.75 ± 0.42	1.06 ± 1.30
*F*		111.87	34.94	15.12	138.04

*Note*. ^*※*^Compared to the control *P* < 0.01; ^▲^compared to the COPD group *P* < 0.01; compared to the ASMCOPD group ^*◆*^*P* < 0.01. *Note*. COPD: chronic obstructive pulmonary disease, ASSCOPD: COPD with abnormal savda syndrome, and ASMCOPD: ASSCOPD treated by abnormal savda munziq.

**Table 5:** Comparison of AQP1 mRNA, AQP4 mRNA, and AQP5 mRNA among groups.

Group	*N*	AQP1 mRNA	AQP4 mRNA	AQP5 mRNA
COPD	5	0.23 ± 0.09^*※※*^	0.62 ± 0.31	0.20 ± 0.08^*※※*^
ASSCOPD	5	0.18 ± 0.08^*※※*^	0.37 ± 0.13^*※※*^	0.25 ± 0.13^*※※*^
ASMCOPD	5	0.43 ± 0.21^*※※*^	0.76 ± 0.35	0.70 ± 0.28^▲*◆*^
Control	5	1.00 ± 0.00	1.00 ± 0.00	1.00 ± 0.00
*F*		64.01	8.35	40.69

*Note*. ^*※*^compared to the control group *P* < 0.05, ^*※※*^*P* < 0.01; ^▲^compared to the ASSCOPD group *P* < 0.05; compared to the COPD group ^*◆*^*P* < 0.05. *Note*. COPD: chronic obstructive pulmonary disease, ASSCOPD: COPD with abnormal savda syndrome, and ASMCOPD: ASSCOPD treated by abnormal savda munziq.

**Table 6:** Comparison of AQP1, AQP4, and AQP5 protein level among groups.

Group	*N*	AQP1	AQP4	AQP5
COPD	5	0.24 ± 0.10^*※※*^	0.24 ± 0.12^*※※*^	0.20 ± 0.07^*※※*^
ASSCOPD	5	0.32 ± 0.13^*※※*^	0.33 ± 0.17^*※※*^	0.16 ± 0.09^*※※*^
ASMCOPD	5	0.73 ± 0.24^*※※*▲*◆*^	0.84 ± 0.15^▲*◆*^	0.59 ± 0.16^*※※*▲*◆*^
Control	5	1.07 ± 0.15	0.97 ± 0.19	1.00 ± 0.19
*F*		27.94	25.35	41.34

*Note*. ^*※*^Compared with control group *P* < 0.05, ^*※※*^*P* < 0.01; ^▲^compared to the ASSCOPD group *P* < 0.01; compared to the COPD group ^*◆*^*P* < 0.01. COPD: chronic obstructive pulmonary disease, ASSCOPD: COPD with abnormal savda syndrome, and ASMCOPD: ASSCOPD treated by abnormal savda munziq.

## References

[1] Zhong N. S., Wang C., Yao W. Z. (2007). Prevalence of chronic obstructive pulmonary disease in China: a large, population-based survey. *The American Journal of Respiratory and Critical Care Medicine*.

[2] Yoo K. H., Kim Y. S., Sheen S. S. (2011). Prevalence of chronic obstructive pulmonary disease in korea: the fourth korean national health and nutrition examination survey, 2008. *Respirology*.

[3] Buist A. S., McBurnie M. A., Vollmer W. M. (2007). International variation in the prevalence of COPD (the BOLD study): a population-based prevalence study. *The Lancet*.

[4] (2011). Global strategy for the diagnosis, management, and prevention of chronic obstructive pulmonary disease. *GOLD Executive Committee*.

[5] Ayinuer M., Yang Y., Halmurat U. (2011). Screening of quantiative indices of typing abnormal hilit syndrome in Uyghur medicine using Delphi method to survey on chronic obstructive pulmonary disease of Xinjiang. *Journal of Xinjiang Medical University*.

[6] Wang H., Gao W., Kong M. (2015). Effects of abnormal savda munzip on the proliferation activity and migration ability of fibroblasts derived from hypertrophic scar in vitro. *Evidence-Based Complementary and Alternative Medicine*.

[7] Upur H., Yusup A., Umar A. (2011). Abnormal savda munziq, an herbal preparation of traditional uighur medicine, may prevent 1,2-dimethylhydrazine-induced rat colon carcinogenesis. *Evidence-Based Complementary and Alternative Medicine*.

[8] Kozono D., Yasui M., King L. S., Agre P. (2002). Aquaporin water channels: atomic structure and molecular dynamics meet clinical medicine. *Journal of Clinical Investigation*.

[9] Ma T., Fukuda N., Song Y., Matthay M. A., Verkman A. S. (2000). Lung fluid transport in aquaporin-5 knockout mice. *Journal of Clinical Investigation*.

[10] Saadoun S., Papadopoulos M. C., Hara-Chikuma M., Verkman A. S. (2005). Impairment of angiogenesis and cell migration by targeted aquaporin-1 gene disruption. *Nature*.

[11] Verkman A. S. (2012). Aquaporins in clinical medicine. *Annual Review of Medicine*.

[12] Jiao G., Li E., Yu R. (2002). Decreased expression of AQP1 and AQP5 in acute injured lungs in rats. *Chinese Medical Journal*.

[13] Hansel N. N., Sidhaye V., Rafaels N. M. (2010). Aquaporin 5 polymorphisms and rate of lung function decline in chronic obstructive pulmonary disease. *PLoS ONE*.

[14] Gao Z., Li F., Upur H. (2014). Effect of cold-dryness on pulmonary and immunologic function in chronic obstructive pulmonary disease model rats. *Journal of Traditional Chinese Medicine*.

[15] Ablimit A., Kühnel H., Strasser A., Upur H. (2013). Abnormal savda syndrome: long-term consequences of emotional and physical stress on endocrine and immune activities in an animal model. *Chinese Journal of Integrative Medicine*.

[16] Zhao W., Sun G. (2011). Exchange of drug dosage between different kinds of experimental animals. *Chinese Journal of Animal Husbandry And Veterinary Medicine*.

[17] Diaz E. A., Chung Y., Denise P. L. (2013). Effects of fresh and aged traffic-related particles on breathing pattern, cellular responses, and oxidative stress. *Air Quality, Atmosphere and Health*.

[18] Nadel J. A. (2013). Mucous hypersecretion and relationship to cough. *Pulmonary Pharmacology and Therapeutics*.

[19] Weiss S. T. (2010). What genes tell us about the pathogenesis of asthma and chronic obstructive pulmonary disease. *American Journal of Respiratory and Critical Care Medicine*.

[20] Caramori G., Di Gregorio C., Carlstedt I. (2004). Mucin expression in peripheral airways of patients with chronic obstructive pulmonary diseasee. *Histopathology*.

[21] Lai Y., Li W. (2009). Expression and clinical significance of mucoprotein MUC5AC in patients with chronic obstructive pulmonary disease. *Acta Medicinae Universitatis Scientiae et Technologiae Huazhong*.

[22] Ma R., Wang Y., Cheng G. (2005). MUC5AC expression up-regulation goblet cell hyperplasia in the airway of patients with chronic obstructive pulmonary disease. *Chinese Medical Sciences Journal*.

[23] Verkman A. S. (2002). Aquaporin water channels and endothelial cell function. *Journal of Anatomy*.

[24] Towne J. E., Harrod K. S., Krane C. M., Menon A. G. (2000). Decreased expression of aquaporin (AQP)1 and AQP5 in mouse lung after acute viral infection. *American Journal of Respiratory Cell and Molecular Biology*.

[25] Sun X., Zhang L., Wu S. (2012). Value of AQP4 expression to explain the significance of the body fluid of lung and the body fluid of large intestine in the pathological mechanism of disease on lung affecting large intestine. *Journal of Emergency in Traditional Chinese Medicine*.

[26] Ziqiang Z. Novel roles of aquaporin 5 in lung cancer and pulmonary epithelial defense function against *P. aeruginosa*.

[27] Krane C. M., Fortner C. N., Hand A. R. (2001). Aquaporin 5-deficient mouse lungs are hyperresponsive to cholinergic stimulation. *Proceedings of the National Academy of Sciences of the United States of America*.

[28] Zhongmin S., Bo Z., Lan Y. (2008). Effect of AQP4 expression on chronic obstructive pulmonary disease. *Journal of Xi'an Jiaotong University (Medical Sciences)*.

[29] Wang K., Feng Y.-L., Wen F.-Q. (2007). Decreased expression of human aquaporin-5 correlated with mucus overproduction in airways of chronic obstructive pulmonary disease. *Acta Pharmacologica Sinica*.

[30] Chunling D., Jihong L., Guifang W. (2007). Expression of AQP5 and MUC5AC and their relationship in lung in rat asthma model. *China Journal of Modern Medicine*.

[31] Chen Z., Zhu R., Bai L., Bai C. (2006). Downregulation of aquaporin 5 induced by vector-based short hairpin RNA and its effect on MUC5AC gene expression in human airway submucosal gland cells. *Respiratory Physiology and Neurobiology*.

[32] Jiao G., Li E., Yu R. (2002). Decreased expression of AQP1 and AQP5 in acute injured lung in rats. *Chinese Medical Journal*.

[33] Yongmin L., Yikemu S. *Uygur Material Medica*.

[34] Ulbricht C., Seamon E., Windsor R. C. (2011). An evidence-based systematic review of cinnamon (*Cinnamomum* spp.) by the natural standard research collaboration. *Journal of Dietary Supplements*.

[35] Huang S.-F., Cheng S.-D., Chuang W.-Y. (2012). Cyclin D1 overexpression and poor clinical outcomes in Taiwanese oral cavity squamous cell carcinoma. *World Journal of Surgical Oncology*.

[36] Seven G., Christoph K., Andreas E. (2012). Expression of p53, p21 and cyclin D1 in penile cancer: p53 predicts poor prognosis. *Journal of Clinical Pathology*.

[37] Jian W., Zhiwei X., Can Y. (2005). Mechanism of phlegm syndrome from pathological changes of mucus-like materials. *Shanghai Journal of Traditional Chinese Medicine*.

